# Supplementary material for: Patterns of Intron Gain and Loss in Fungi
Source: PLoS Biol. 2004 Nov 30;2(12):e422. doi: 10.1371/journal.pbio.0020422 (PMC532390; doi:10.1371/journal.pbio.0020422)
Supplement: Table S1 — Also available at http://genes.mit.edu/NielsenEtAl/. (4.3 MB ZIP). [file pbio.0020422.st001.zip › NielsenEtAl/html/746.html]

AN3642.1.NCU02743.1.MG04830.1.FG01155.1


```
 CLUSTAL W (1.82) Multiple Sequence Alignments - Introns Inserted


Sequence 1: NCU02743.1	327 aa
Sequence 2: FG01155.1	318 aa
Sequence 3: MG04830.1	317 aa
Sequence 4: AN3642.1	347 aa
Alignment Length: 347 aa
Number Identitical Residues: 264 aa
Alignment Score (without introns) 10326


MG04830.1 	-MSYFFSTPVDIDIVLEDGDDRSMVDIKLDKNRREKAPLYMDGESVKGAVTVRPKDGKRL
NCU02743.1	-MSYFFATPVDIDIVLDDADERSMVDVKLDKNRREKAPLYMDGESVKGAVTVRPKDGKRL
FG01155.1 	-MSYFFATPVDIDVVLDDTDDRSMVDVKLDKNRREKAPLFMDGESVKGAVTVRPKDGKRL
AN3642.1  	MTSLFFSTPLDIDIVLEDGDERQMVDVKLDKGHREKVPLYMDGESVKGAVTVRPKDGKRL
          	  * **:**:***:**:* *:*.***:****.:***.**:********************

MG04830.1 	EHTGIKVQFIGSIE~MFFDRGNHYEFLSLVQELAAPGELQHPQTFDFNFKNVEKQYESYN
NCU02743.1	EHTGIKVQFIGTIE~MFFDRGNHYEFLSLVQELAAPGELQHPQTFDFNFRNVEKQYESYN
FG01155.1 	EHTGIKVQFIGTIE1MFFDRGNHYEFLSLNQELAAPGELQHPQTFDFNFKNVEKQYESYN
AN3642.1  	EHTGIKVQFIGTIE1IFYDRGNHHEFLSLVQELAAPGELQHPQTFPFNFKNVEKQYESYN
          	***********:** :*:*****:***** *************** ***:**********

MG04830.1 	GINVKLRYFVRVTVSRRMADVIREKDIWVYSYRIPPEINSSIKMDVGIEDCLHIEFEYSK
NCU02743.1	GINVKLRYFVRVTVSRRMADVIREKDIWVYSYRIPPEMNSSIKMDVGIEDCLHIEFEYSK
FG01155.1 	GINVKLRYFVRVTVSRRMADVIREKDIWVYSYRIPPEMNSSIKMDVGIEDCLHIEFEYSK
AN3642.1  	GINVKLRYFVRVTVSRRMADVIREKDLWVYSYRMPPETNSPIKMDVGIEDCLHIEFEYSK
          	**************************:******:*** **.*******************

MG04830.1 	SKYHLKDVIVGRIYFLLVRLKIKHMELSIIRRETTGAAPNQYNESETLVRFE0IMDGSPS
NCU02743.1	SKYHLKDVIVGRIYFLLVRLKIKHMELSIIRRETTGVAPNQYNESETLVRFE0IMDGSPS
FG01155.1 	SKYHLKDVIVGRIYFLLVRLKIKHMELSIIRRETTGAAPNQYNESETLVRFE~IMDGSPS
AN3642.1  	SKYHLKDVIVGRIYFLLVRLKIKHMELSIIRRETTGTRPNEYNESETLVRFE0IMDGSPS
          	************************************. **:*********** *******

MG04830.1 	RG~ETIPIRLFLGGFDLTPTFRDVNKKFSTRYYLSLVLIDED1ARRYFKQSEIILYRQTP
NCU02743.1	RG~ETIPIRLFLGGFDLTPTFRDVNKKFSTRYYLSLVLIDED1ARRYFKQSEIILYRQAP
FG01155.1 	RG~ETIPIRLFLGGFDLTPTFRDVNKKFSTRYYLSLVLIDED1ARRYFKQSEIILYRQAP
AN3642.1  	RG1ETIPIRLFLGGFDLTPTFRDVNMKFSTRYYLSLVLIDED1ARRYFKQSEIILHRLAP
          	** ********************** **************** ************:* :*

MG04830.1 	QELNG-----------SQQLP----APPE-----------SRIQAVPA---
NCU02743.1	EIAAA-----------AAAAPPGQNALPAPPPND------NRLAGIPAA--
FG01155.1 	DAAPV-----------LNAG---TSSLPE------------NKAVIAQA--
AN3642.1  	EIPPTPEVAKQQQIQMQQAAEQQQHQLPPGSATAGPGREHVNSQAVPPPAA
          	:    .. :.... .            *  ... ... .  .   :. .::
```
